# Breast Cancer Resistance to Cyclin-Dependent Kinases 4/6 Inhibitors: Intricacy of the Molecular Mechanisms

**DOI:** 10.3389/fonc.2021.651541

**Published:** 2021-05-26

**Authors:** Bin Wang, Rui Li, Shuai Wu, Xin Liu, Jianlin Ren, Jing Li, Kaixin Bi, Yanhong Wang, Hongyan Jia

**Affiliations:** ^1^ Department of Breast Surgery, First Hospital of Shanxi Medical University, Taiyuan, China; ^2^ Department of Microbiology and Immunology, Shanxi Medical University, Taiyuan, China

**Keywords:** breast cancer, CDK4/6 inhibitors, drug resistance, molecular mechanisms, combination administration

## Abstract

Breast cancer is a common malignant tumor in women, with a highest incidence and mortality among all of the female malignant tumors. Notably, targeted therapy has achieved impressive success in the treatment of breast cancer. As one class of the anti-tumor targeted therapeutics, Cyclin-Dependent Kinases 4/6CDK4/6inhibitors have shown good clinical activity in treating breast cancer. Nevertheless, despite the promising clinical outcomes, intrinsic or acquired resistance to CDK4/6 inhibitors has limited the benefits of this novel target therapy. In the present review, we provide an overview of the currently known molecular mechanisms of resistance to CDK4/6 inhibitors, and discuss the potential strategies to overcoming drug resistance improving the outcomes for breast cancer patients treated with CDK4/6 inhibitors.

## Introduction

Cyclin-dependent kinases (CDKs) are serine/threonine kinases that play key roles in regulating cell cycle (1). CDK 4 and 6, two critical kinases among CDKs mediate the cellular transition from G0/G1 phase to S phase during cell cycle: dysregulation of CDK 4/6, result in uncontrolled cell division. The main effect of CDK4/6 inhibitor is to bind with cyclin D specifically, block cell cycle transformation, and stop cell cycle in G1 phase, thereby inhibiting tumor cell proliferation (2). Importantly, CDK4/6 inhibitors have showed great efficacy in treatment of breast cancer. Based on the PALOMA-1trail, FDA approved palbociclib, the first CDK 4/6 inhibitor, in combination with letrozole as first-line treatment for patients with ER-positive, HER2-negative advanced breast cancer (ABC) or metastatic breast cancer (MBC) (3). At present, three selective CDK4/6 inhibitors (palbociclib, ribociclib, and abemaciclib) have been approved by FDA (4, 5). These three CDK4/6 inhibitors are used in combination with endocrine therapies or fulvestrant for patients with ER+ Her− metastatic breast cancer. Clinical trials PALOMA-2, MONALEESA-2, and MONARCH-3 have showed that when combined with aromatase inhibitors, CDK4/6 inhibitors could significantly prolong the progression-free survival in postmenopausal women with HR-positive metastatic breast cancer (6–8). Nevertheless, despite promising clinical outcomes, acquired or intrinsic resistance to CDK4/6 inhibitors often occurs, and this constitutes a major hindrance to successful treatment and limits the therapeutic benefits of those targeted therapeutics for patients with this disease. Therefore, understanding the molecular mechanisms and pathways involved in resistance to CDK4/6 inhibitors may help develop effective strategies to circumventing drug resistance and selecting patient populations who can benefit from this targeted therapy. Here, we review and discuss the known molecular mechanisms and pathways that modulate the cellular sensitivity or resistance to CDK4/6 inhibitors, and provide our outlook on this subject (6–8).

## Potential Resistance Mechanisms

**Figure 1:**
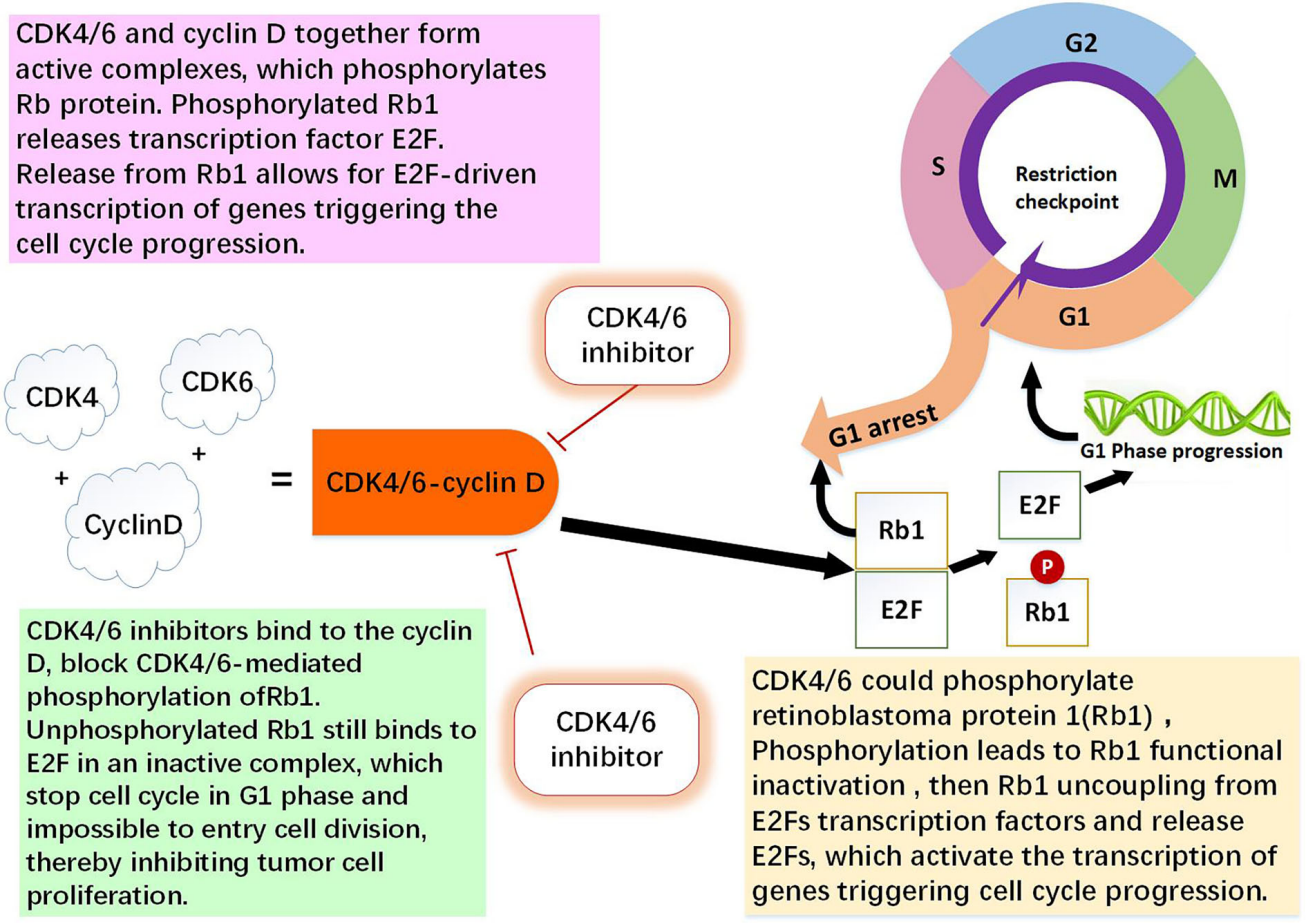
The role of CDK4/6-cyclin D and CDK4/6 inhibitor. CDK4 and CDK6 play key roles in cell proliferation. Cyclin D is regulator of the CDK4 and CDK6 kinases. CDK4/6 and cyclin D together form active complexes, which phosphorylates Rb1 protein. Rb1 is an onco-suppressor which repress the transcription of genes required for the cell cycle, limit the expression of transcription factor E2F target genes which are involved in cell cycle progression. Phosphorylated Rb1 releases E2F. Release from Rb1 allows for E2F-driven genes triggering the cell cycle progression. CDK4/6 inhibitors bind to the cyclin D specifically, thereby block CDK4/6-mediated phosphorylation of Rb1. Non-phosphorylated Rb1 still binds to E2F in an inactive complex, which leading to cell cycle arrest in G0/G1 phase and impossible to entry cell division, thereby inhibiting tumor cell proliferation (1, 9, 10, 13).

Breast cancer cells can be intrinsically resistant to CDK4/6 or develop acquired resistance to those agents. CDK4/6 can phosphorylate retinoblastoma protein (Rb1), and the phosphorylation leads to Rb1 functional inactivation, then Rb1 uncoupling from E2Fs transcription factors and release E2Fs. CDK4/6 inhibitors exert their effects through breaking the CDK4/6-Rb-E2F pathway (9, 10). The tumor cells with loss of Rb1 and lack of the major targets, intrinsic resistance to CDK4/6 inhibitors may occur (11, 12). The major obstacle to successful treatment with CDK4/6 inhibitors is the acquired resistance that frequently occurs in the patients who have received this therapy. Tumor cells can acquire the ability to escape CDK4/6 action (13). Understanding potential mechanisms of acquired resistance to CDK4/6 inhibitors may help find effective ways to preventing or overcoming drug resistance to this class of therapeutics (**Figure 1**).

## Direct Cell Cycle Mechanisms

### Loss of Drug Target Genes

#### RB1

**Figure 2:**
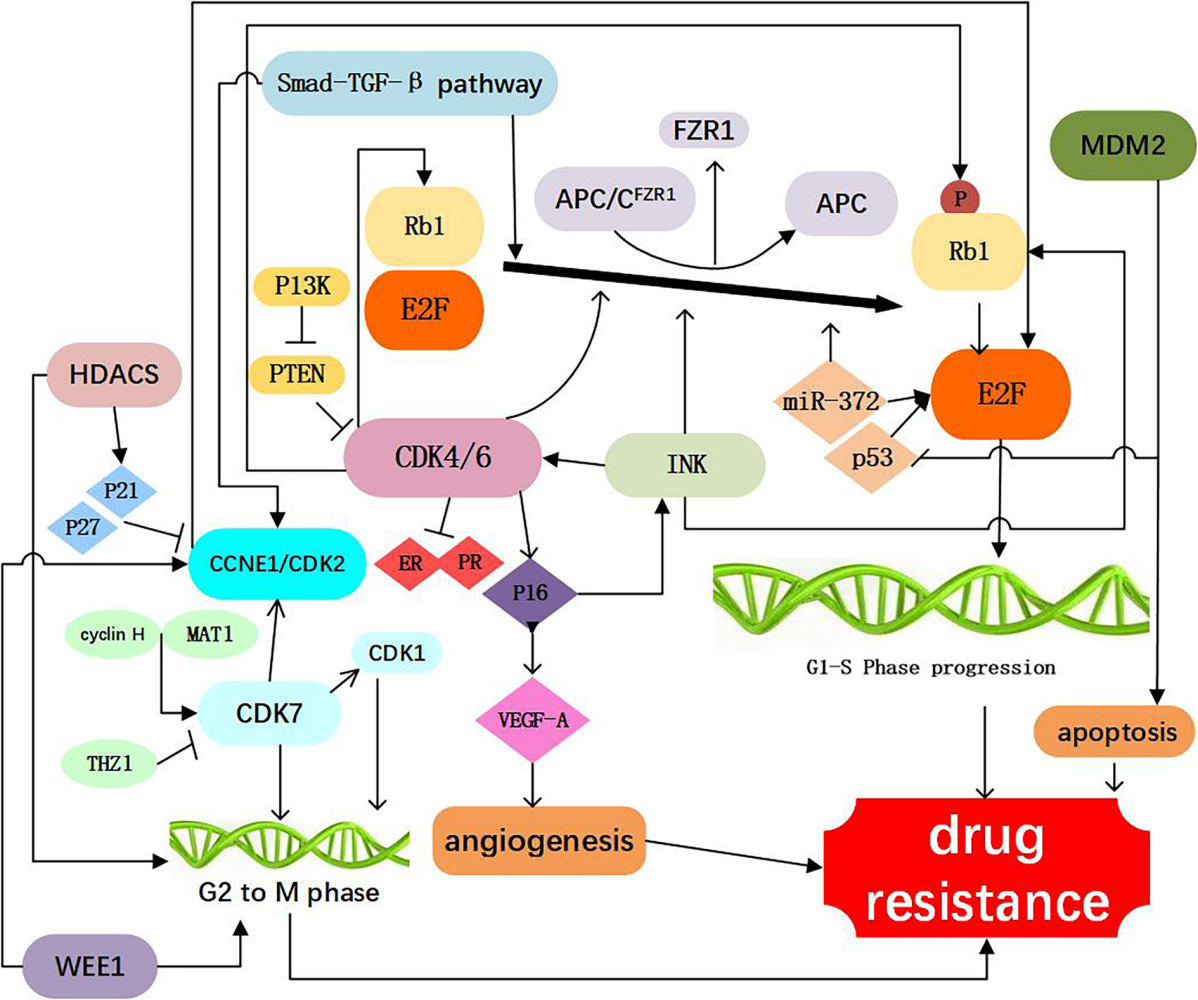
Resistance to CDK4/6 inhibitors: Direct Cell Cycle Mechanism: 1. Loss of drug target genes: APC/CFZR1 promote the phosphorylation of Rb1 and regulate cell transition from G1 to S. knockdown of Rb1 and FZR1 synergistically bypassed cell division arrest induced by the CDK4/6 inhibitor (14–23); 2. Increased activity of the CDK4 and CDK6: amplification of CDK4/6 account for a decreased CDK4/6 targeted phosphorylation of Rb1 and a decreased sensitivity of breast cancer cells to CDK4/6 inhibitor (2, 24–34); 3. Abnormal regulations of upstream and Downstream kinases: CCNE1/CDK2, CDK7, E2F, INK, PTEN, Smad-TGF-β pathway which are involved in the progression of cell cycle, as shown in **Figure 1**, are responsible for resistance to CDK4/6 inhibitors (12, 15, 17, 26, 35–60); 4. Activation of alternate genes like HDACS, WEE1, MDM2, partly help the cancer cell escape from the drugs work (61–67).

The tumor suppressor Rb1 is a key checkpoint in the cell cycle and a major target of CDK4/6 inhibitors. In both of preclinical and clinical settings, Rb1 mutations were found (14). In the tumor cell line with acquired resistance to palbociclib, it was demonstrated that resistance to CDK4/6 inhibitors was mediated through Rb1 loss, and restoration of Rb1 expression rendered tumor cells sensitivity to the CDK4/6 inhibitor (15). Chronic loss of Rb1 was found to be a cause of resistance to CDK4/6 inhibitors in breast cancer (16, 17). Using the breast cancer cell lines sensitive or resistant to palbociclib, it was showed that the complex change of Rb1 pathway was related to resistance to CDK4/6 inhibitor, Rb1 deficient in function is an important factor that contributes to palbociclib and abemaciclib resistance in breast cancer patients (18, 19). In clinical settings, researchers sequenced the somatic genomic mutations of three HR+ breast cancer samples before and after drug resistance to CDK4/6 inhibitors occurred and found that Rb1 mutation, allele substitution or exon deletion only existed in the blood samples after but not before drug resistance (20). Many researches showed that Rb1 loss could activate bypass of cyclin D1-CDK4/6-dependent pathway, leading to acquired resistance to CDK4/6 inhibition (14). These observations suggest that despite loss of Rb1, progression of the cell cycle continues *via* the activation of other cell cycle machinery, and inhibition of the bypass axis in combination with the CDK4/6 inhibitors may be effective in overcoming resistance to these targeted therapies. However, in the PALOMA-3 randomized phase III trial, the circulating tumor DNA sequencing from patients showed that Rb1 mutations occurred only in 6 of 127 (4.7%) patients (21). Thus, further clinical evidence is needed to analyze the frequency of Rb1 mutation in breast cancer patients receiving CDK 4/6 treatment (**Figure 2**, **Table 1**).

**Table 1:** Mechanisms of acquired resistance to CDK4/6 inhibitors: Direct cell cycle mechanisms.

Resistance classify	Resistance mechanism	Detection	Overcome
Loss of drug target genes (14–23)	Loss of Rb1	1. Cell biology experiments	1. Restore Rb1 expression
		2. Proteomics	
		3. Clinical trial	2. Bypass way
	Loss of APC/C^FZR1^	1. Cell biology experiments	1. Restore FZR1 expression
Increased activity of the target genes (2, 24–34)	CDK4 amplification	1. Cell biology experiments	1. Knockdown of CDK4
		2. Proteomics	
		3. Immunohistochemistry	2. Bypass way
		4. Clinical trial	
	CDK6 amplification	1. Cell biology experiments	1. Knockdown of CDK6
		2. Proteomics	
		3. Immunohistochemistry	2. Bypass way
		4. Clinical trial	
Abnormal regulations of upstream and downstream kinases (12, 15, 17, 26, 35–60)	Increased expression of CCNE1/CDK2	1. Cell biology experiments	1.CDK2 inhibitor
		2. Proteomics	
		3. Immunohistochemistry	2. Bypass way
		4. Chip-seq analysis	
	CDK7 overexpression	1. Cell biology experiments	CDK7 inhibitor
		2. Proteomics	
		3. Immunohistochemistry	
	E2F overexpression	1. Cell biology experiments	1. E2F inhibitor
		2. Proteomics	2. Inhibition regulate gene or protein downstream of E2F
		3. Biopsies mRNA gene expression	
	p16INK4A (p16) overexpression	1. Cell biology experiments	1. Restore p16 expression
		2. Proteomics	2. p16 methylation
	Loss of PTEN	1. Cell biology experiments	1. Restore PTEN expression
		2. Proteomics	
		3. Biopsy	
	Smad-TGF-β pathway dysregulation	1. Cell biology experiments	1. Activate smad3
			2. TGF-β inhibitor
		2. Proteomics	3. Inhibition of EMT
Activation of alternate genes (61–67)	WEE1 overexpression	1. Cell biology experiments	WEE1 inhibitor
		2. Proteomics	
	MDM2 overexpression	1. Cell biology experiments	MDM2 inhibitors
		2. Proteomics	

CDK, Cyclin-dependent kinases; Rb1, Retinoblastoma protein1; APC/C, anaphase promoting complex/cyclosome; PTEN, Phosphatase and tensin homolog; TGF-β, transforming growth factor β; WEE1, serine/threonine kinases gene; MDM2, Mouse double minute 2 homolog.

#### APC/C^FZR1^

Similar to Rb1, the ubiquitin ligase anaphase promoting complex/cyclosome (APC/C) play an important role in cell cycle regulation. APC/C and pRb interact *via* the co-activator of APC/C^FZR1^, providing an alternative pathway to regulate transition from G1 to S by pRb through a post-translational mechanism (22). FZR1 is a candidate CDK4/6-cyclin D substrate and as an important determinant in response to CDK4/6 inhibitors. It was found that the loss of FZR1 resulted in uncontrolled cell cycle progression from G1 to S phase. In human breast cancer cell lines, simultaneous knockdown of Rb and FZR1 synergistically bypassed cell division arrest induced by the CDK4/6 inhibitor PD-0332991 (23). The precise mechanism of resistance to CDK4/6 inhibitors associated with the loss of FZR1 remains unclear. It is likely that loss of FZR1 corresponds with the loss of Rb; however, this possibility remains to be further investigated (**Figure 2**, **Table 1**).

### Increased Activity of the Target Genes

#### CDK4

CDK4 is an important component of the cyclind-CDK4/6-Rb1 pathway, and was observed in 25% luminal B and 14% Luminal A breast cancers (24). In addition, aberrant expression of CDK4 activates the cyclind-CDK4/6-Rb1 pathway and results in drug resistance (25). It has been demonstrated that CDK4 was elevated in palbociclib resistant cell lines (26). Also, amplification of CDK4 has been reported in melanoma, glioma, rhabdomyosarcoma, and lung cancer and confers resistance to CDK4/6 inhibitors in these malignancies (27–30). The researchers found that increasing phosphorylation of p27 could inhibit CDK4 and regulate the cyclin D/CDK4/p27 complex activity, which could make breast cancer cells more resistant to palbociclib (2, 31), above study suggesting a potential strategy to prevent adaptation to CDK4/6 inhibitors (**Figure 2**, **Table 1**).

#### CDK6

The functions of CDK6 are both kinase-dependent and non-kinase-dependent (32). After a prolonged exposure to CDK4/6 inhibitor LY2835219, a significant amplification of CDK6 was found in several breast cancer cell lines, and this may account for a decreased CDK4/6 targeted phosphorylation of Rb1 and a decreased sensitivity of breast cancer cells to CDK4/6 inhibitor (32). Further experiments confirmed that forced overexpression of CDK6 indeed mediated drug resistance. Overexpression of CDK6 not only mediates resistance to CDK4/6 inhibitors, but also leads to decreased expression of estrogen and progesterone receptors. These studies also suggest that the efficacy of CDK4/6 inhibitors in breast cancer cells is modulated by ER. Therefore, CDK6 amplification can decrease the tumor cell sensitivity to both ER antagonists and CDK4/6 inhibitors. Knockdown of CDK6 can restore sensitivity, while enforced overexpression of CDK6 can confer resistance to CDK4/6 inhibitors

A decrease in ER/PR expression was observed in the tumor specimens from patients receiving treatment of CDK4/6 inhibitor and showing insensitivity to CDK4/6 inhibitors (33). The non-kinase dependent function of CD6 lies in its transcriptional regulation function. In the STAT3 and Cyclin D pathways, CDK6 could up-regulate the transcription of P16 and the expression of VEGF-A that can promote angiogenesis, contributing to the progression and drug resistance of breast cancer (32, 34) (**Figure 2**, **Table 1**).

### Abnormal Regulations of Upstream and Downstream Kinases

#### CCNE1/CDK2

The cyclin E (encoded by CCNE1 gene)-CDK2 complexes play a key role in the cell cycle from G1 to S phase. Cyclin E-CDK2 can phosphorylate Rb1, release E2F, and promote entry into the S phase (35, 36). In an analysis of global gene expressions, increased expression of CDK2 was found in the palbociclib-resistant breast cancer cell lines. Also it was suggested that loss of p21 and p27, which has an inhibitory effect function on CDK2, may represent a mechanism leading to bypass of palbociclib (17). It has been reported that when combined CDK2 and CDK4 inhibitors, resistance to palbociclib was no longer obvious, suggesting that cyclin E-CDK2 complexes protein might mediate resistance to CDK4/6 inhibitors (37). Hopefully, next generation CDK inhibitors can target CDK2 to prevent or conquer drug resistance (**Figure 2**, **Table 1**).

#### CDK7

CDK7, one of the major cell cycle regulators, acts as a CDK-activating kinase (CAK) by maintaining CDK1 and CDK2 activity. CDK7 promotes the cell transition from G2 phase to M phase (38). It has been demonstrated that CDK7 overexpression occurred in the estrogen receptor-positive, palbociclib-resistant breast cancer cells (26), suggesting that CDK7 is involved in cellular resistance to CDK4/6 inhibitors. The CDK7 selective inhibitor, THZ1, can significantly inhibit the proliferation of triple negative breast cancer cells at the nmol/L concentration (39, 40). Also, the sensitivity of breast cancer cells to CDK7 inhibitors appears to be associated with the loss of ER and Rb1 CN expression (26). Thus, CDK7 inhibitors may play an important role in both of the targeted therapy and cellular resistance to CDK4/6 inhibitors (**Figure 2**, **Table 1**).

#### E2F

The CDK-Rb-E2F pathway plays a critical role in the control of cell cycle in breast cancer. At the early stage of G1, E2F binds to Rb1 protein and forms a functional complex. Phosphorylation of Rb1 protein by CDK activates E2F. Activation of E2F can promote the transition of cells from G1 phase to S phase. It has been reported that in the CDK4/6 inhibitor-resistant cell lines, the CDK-Rb-E2F pathway reactivate (41). Researchers found that in tumor biopsies resistant to palbociclib, CCND3, CCNE1, and CDKN2D are persistently elevated before palbociclib used, all three genes are known E2F1 transcription targets, suggesting persistent E2F activity in resistant tumors (42). It was also revealed that E2F1 was up-regulated in patients with tumor lymph node metastasis and advanced stage (43) and patients with increased E2F expression was associated with lower overall survival (OS), relapse-free survival (RFS), distant metastasis-free survival (DMFS) (44). Therefore, E2F might be exploited as a therapeutic target both for suppressing drug resistance to CDK4/6 inhibitors and biomarkers and therapeutic targets for breast cancer in breast cancer.

#### INK

CDK4/6 activity is regulated by the INK4 family proteins (p16INK4A, p15INK4B, p18INK4C, and p19INK4D), can inhibit the expression of CDK4 and lead to cell cycle arrest in the G1/S phase, thus considered as a natural tumor inhibitor (45). The P16 (p16INK4A) protein, encoded by the CDKN2A^ink4a^ gene, play an important role of the INK4 family. It has been reported that CDK4/6 inhibitors can inhibit cancer cell cycle progression because of P16 gene deletion (46). Cancer cells with P16 methylation are more sensitive to palbociclib than those control (47, 48). It has been found that overexpression of p16 and loss of Rb1 often occur simultaneously. When p16 overexpression is accompanied by Rb1 deficiency, CDK4/6 inhibitors are inactive due to the Rb1 deficiency. With the presence of Rb1, overexpression of p16 (be consistent) leads to a decrease of CDK4 and resistance to CDK4/6 inhibitors (12). Further studies are needed to delineate the precise mechanistic association between Rb1 loss and P16 overexpression, which may help design novel therapeutic strategies to overcoming the acquired resistance to CDK4/6 inhibitors (**Figure 2**, **Table 1**).

#### PTEN

PTEN a tumor suppressor gene, is one of the frequently mutated genes in human cancers (49). The increased expression of PTEN leads to the inactivation of CDK, which enables the Rb1 keep dephosphorylating, while binding to transcription factor E2F, which ultimately inhibits cell proliferation. these ways may influence the effect of CDK4/6 inhibitors (49). Researchers analyzed serial biopsies from breast cancer patients treated with the combination of ribociclib and letrozole and found that ablation of PTEN was sufficient to promote resistance to CDK4/6 inhibition (50). The increased AKT expression could reduce PTEN expression and render breast cancer cells resistant to CDK4/6 inhibitors (51). In breast cancer cells, loss of PTEN also conferred resistance to alpelisib. Moreover, loss of PTEN expression can cause dual resistance to CDK4/6 inhibitors and PI3K inhibitors (52) (**Figure 2**, **Table 1**).

#### Smad-TGF-β Pathway

Smad–transforming growth factor β (TGF-β) pathway contributes to G1 arrest in breast cancer cells (53). TGF-β signaling is transduced through Smad2 and Smad3 and forms a complex with Smad4 to regulate target gene expression relevant to cell growth and differentiation (54, 55). Smad3, which has antiproliferative effects, has a key role in TGF-β signaling cascade. Smad3 can regulate cell cycle arrest, and has been shown to be correlated with resistance to CDK4/6 inhibitors (53). Mechanistically, cyclin E-CDK2 and cyclin D1-CDK4/6 complexes can suppress Smad3 through its phosphorylation, and the suppression of Smad3 releases the Rb1-E2F blockade and restore cell cycle arrest in breast cancer cells (53, 56). TGF-β can phosphorylate and activate Smad2 and Smad3 and form a complex with Smad4, and this contributes to the induction and progression of EMT. EMT can promote invasion and metastasis of cancer cells and increase drug resistance (57). Consistently, inhibition of the CDK2-mediated phosphorylation of Smad3 reduces TNBC cell migration and invasion through changes in EMT-related signaling factors (58). According to these findings, resistance of tumor cells to CDK4/6 inhibitors may result from suppression of Smad3 that is associated with the activated cyclin E-CDK2 axis and EMT (15, 36, 59, 60). Thus, the Smad-TGF-β pathway might be considered as a potential therapeutic target for overcome drug resistance to CDK4/6 inhibitors (**Figure 2**, **Table 1**).

### Activation of Alternate Genes Are Involved in the Progression of Cell Cycle

#### WEE1

WEE1 is a protein tyrosine kinase that phosphorylates CDK1 and CDK2 and causes their inhibition (61). WEE1 inhibits CDK1 to maintain the cell in an inactive state and prevent mitosis. WEE1 also inhibits CDK2 to delay the replication process and allow time for DNA repair. Both of these events occur in breast cancer cells (61, 62). Inhibiting the expression of WEE1 can sensitize the drug resistant cancer cells to CDK4/6 inhibitors, probably because that inhibiting WEE1 can increase the expression of CD4 (63). In the ribociclib-resistant cancer cells, a down-regulation of the G2/M checkpoint was observed (64). Drug resistant cancer cells exhibited collateral sensitivity to the Wee-1 inhibitor, adavosertib (AZD1775). Combined treatment with ribociclib and adavosertib can elicit significantly stronger antiproliferative effect on drug resistant tumor cells cells than ribociclib alone (64) (**Figure 2**, **Table 1**).

#### MDM2

Mouse double minute 2 homolog (MDM2) is a negative regulatory protein of tumor suppressor p53 and can inhibit cellular senescence. MDM2 binds to p53 protein and inhibits the function of this tumor suppressor (65). Overexpression of MDM2 drives breast oncogenesis and blocks apoptosis of breast cancer cells, resulting in resistance of tumor cells to CDK4/6 inhibitors. Therefore, the use of MDM2 inhibitors may reverse cellular resistance to CDK4/6 inhibitors, and this has been in human liposarcoma (66). Indeed, the MDM2 inhibitor, CGM097, in combination with a CDK4/6 inhibitor palbociclib and fulvestrant has shown promising therapeutic benefits in reversing the tumor resistance to CDK4/6 inhibitors and to endocrine therapy (67) (**Figure 2**, **Table 1**).

## Indirect Cell Cycle Mechanisms

### Bypass Pathways of the Cell Cycle

#### mTOR Pathway

**Figure 3:**
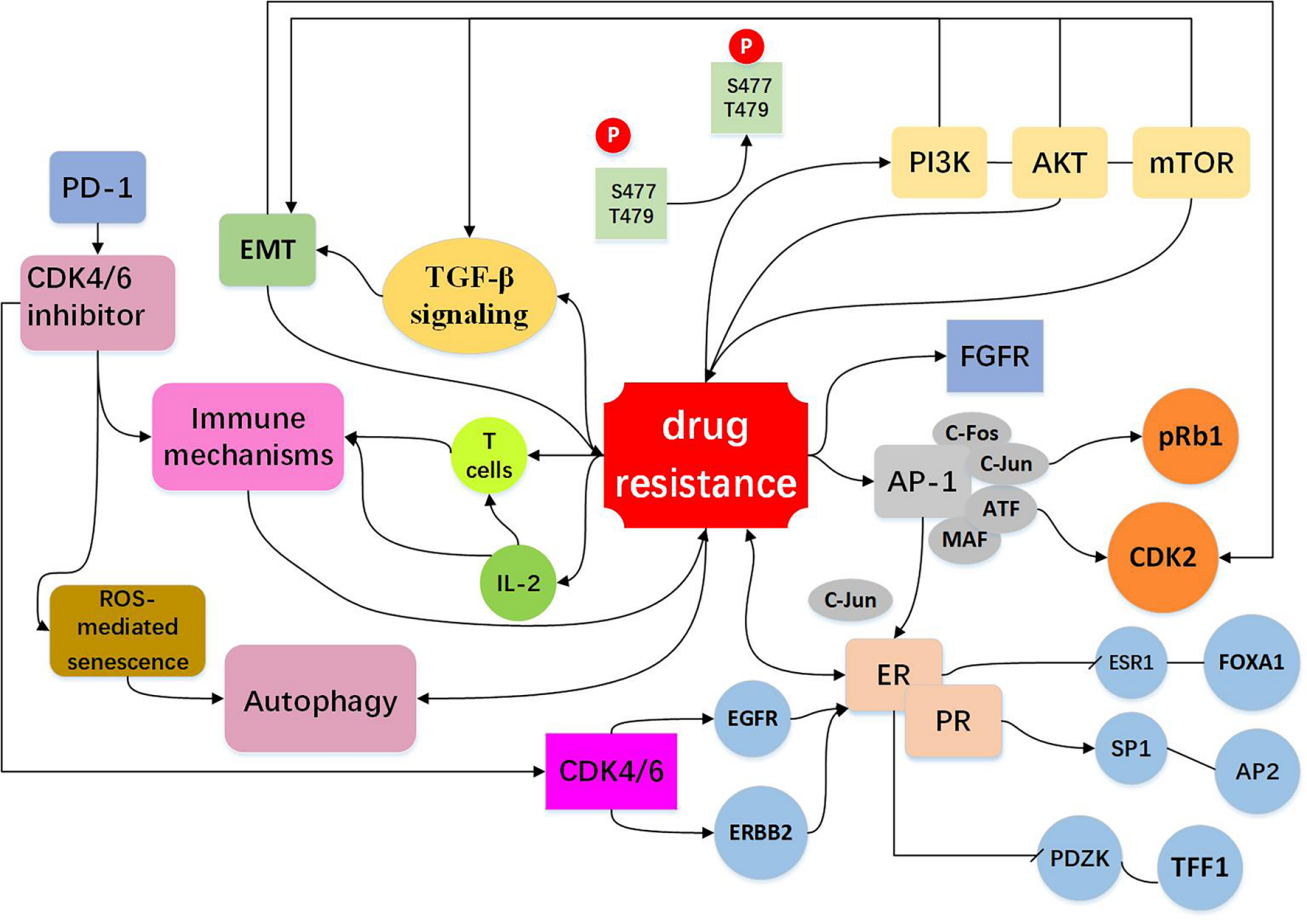
Resistance to CDK4/6 inhibitors: Indirect Cell Cycle Mechanism Bypass pathways of the cell cycle: mTOR activation is *via* phosphoinositide PI3K/AKT signaling. The PI3K/AKT/mTOR pathway regulate cell signal transduction, have extensive links with other bypasses, for example EMT and TGF-β pathway (15, 41, 68–74). High expression of AP-1 (75–78), FGFR amplification (79–82), loss of ER or PR (13, 16, 26, 72) expression drives cells to escape CDK4/6 inhibition and act as bypass pathways for the progression of the cell. Other mechanisms include EMT (10, 83–87), immune mechanisms (88–91) and autophagy directly or indirectly influence drug resistance shown in the figure (10, 92–96).

Abnormal activation of mammalian target of rapamycin (mTOR) pathway is an important target for development of anti-cancer drug, the most common mechanism of mTOR activation in breast cancer is *via* phosphoinositide 3-kinase (PI3K)/protein kinase B (AKT) signaling, PI3K/AKT/mTOR pathway is closely associated with cellular resistance to CDK4/6 inhibitors (15, 68–70). It was reported that mTOR signaling is dysregulated in breast cancer patients following abemaciclib treatment (70) and PI3K/mTOR pathway has been shown to be upregulated in response to chronic exposure to CDK4/6 inhibitors (71). Also, pre-treatment with mTOR inhibitors was shown to prevent or delay the resistance to CDK4/6 inhibitors (72). In a kinome-wide siRNA screen, it was found that the AKT pathway is highly activated in the ribociclib resistant breast cancer cells (73). Combination of PI3K and CDK 4/6 inhibitors could reduce cell viability and overcome intrinsic and adaptive resistance leading to tumor regressions (74). Further studies demonstrated that ribociclib in combination with an AKT inhibitor or PI3K inhibitor has a significantly stronger inhibitory effect on the growth of transplanted tumor in mouse models, as compared with ribociclib alone, supporting the role of PI3K signaling pathway in mediating resistance to the CDK4/6 inhibitor (73). Thus, coinhibition of the PI3K/mTOR and CDK4/6 pathways may prevent induction of drug resistance. Furthermore, it has been showed in a preclinical model that a PI3K inhibitor combined with a CDK4/6 inhibitor has a significant stronger inhibitory effect on proliferation of breast cancer cells than the single drug (41). Taken together, the PI3K/AKT/mTOR inhibitors may represent a class of sensitizers in CDK4/6-targeted therapy (**Figure 3**, **Table 2**).

**Table 2:** Mechanisms of acquired resistance to CDK4/6 inhibitors: Indirect cell cycle mechanisms.

Resistance classify	Resistance mechanism	Detection	Overcome
Bypass pathways of the cell cycle (13, 15, 16, 26, 41, 68–82)	mTOR pathway	1. Clinical trial	1. mTOR inhibitor
		2. Cell biology experiments	2. AKT inhibitor
		3. Immunohistochemistry	3. PI3K inhibitor
		4. Animal model	
	High expression of AP-1	1. Clinical trial	1. AP-1 inhibitor
		2. Cell biology experiments	
		3. Immunohistochemistry	
	FGFR amplification	1. Clinical trial	1. Anti-FGFR drug
		2. Cell biology experiments	
		3. Immunohistochemistry	
	Loss of ER or PR expression.	1. Preliminary clinical study	1. ER regulator/blocker
		2. Chip-seq analysis	2. Bypass way
		3. Cell biology experiments	
Other mechanisms (10, 83–96)	EMT	1. Gene set enrichment analysis (GSEA)	1. Inhibition of EMT
		2. Proteomics	
		3. Immunohistochemistry	2. Bypass way
		4. Cell biology experiments	
	Immune mechanisms	1. Proteomics	1. Immune checkpoint inhibitors
		2. Experimental animal models	
		3. Cell biology experiments	2. Immunotherapy
	Autophagy	1. Proteomics	1. Autophagy inhibitor
		2. Immunohistochemistry	2. Autophagy proteins

PI3K, phosphatidylinositide 3-kinases; AKT, protein kinase B;mTOR, mammalian target of rapamycin; AP-1, Activator protein 1; ER, estrogen receptor;

PR, progesterone receptor; FGFR, fibroblast growth factor; EMT, Epithelial-mesenchymal transformation; receptor.

#### AP-1

High expression of AP-1 can lead to resistance to CDK4/6 inhibitors. AP-1 family consists of C-FOS, C-Jun, ATF, and MAF, and is involved in the regulation of a variety of genes, including cyclinD (75). The high expression of C-Jun is common in breast cancer and affects the expression of ER (76). It was found in breast cancer cells that are resistant to palbociclib which the transcriptions of AP-1 and C-FOS were increased, and AP-1 blockade in combination with palbociclib could effectively inhibit cell proliferation and reduce pRb and CDK2 levels as compared to single agent treatment (77). These observations suggest that co-treatment with Ap-1 specific inhibitors and CDK4/6 inhibitors may elicit anti-tumor synergistic effects. AP-1 and c-FOS inhibitors have entered Phase II clinical trial (T-5224) (78) (**Figure 3**, **Table 2**).

#### FGFR

The fibroblast growth factor receptor (FGFR) is growth factor receptor tyrosine kinases (79). Development of normal mammary gland requires active transcription of FGFR mediated proto-protein kinase and FGFR is closely associated with the development and progression of breast cancer (80, 81). Based on the combination of letrozole with ribociclib, the clinical trial MONALESA-2 observed that FGFR1 amplification was related to a lower PFS (79). It was also demonstrated that FGFR1 expression was increased in breast cancer MCF-7 cells treated with fulvestrant and palbociclib (82), and lucitanib, an anti-FGFR drug, can decrease drug resistance. As FGFR1 can stimulate the proliferation capacity of cancer cells, inhibiting both FGFR/FGF and the CDK4/6 pathways might be an effective approach to preventing or circumventing resistance to a single agent (**Figure 3**, **Table 2**).

#### ER and PR

ER and PR are the major factors that mediate cyclinD-CDK4/6 activity in estrogen receptor-positive (ER+) and progesterone receptor-positive (PR+) breast cancer cells (13). Effect of ER on resistance to CDK4/6 inhibitors involves both cell cycle and non-cell cycle mechanisms. In a preliminary clinical study, it was found that the expressions of ER/PR were lost in the palbociclib resistant tumor samples and down-regulated in the palbociclib resistant breast cancer cells (16, 26). Chip-seq analysis uncovered that ER was deficient in binding to ESR1 and FOXA1, but enriched in binding to SP1 and AP2, and these were accompanied by decreased expression of regulatory genes such as PDZK1 and TFF1.These data indicate that drug-resistant cells are genetically altered by chromosome remodeling. In other pathways discussed above, high expression of AP-1 leads to overexpression of C-Jun, which inhibits ER activity and modulates the efficacy of CDK4/6 inhibitors (76). Similarly, CDK4/6 blockade can lead to up-regulation of EGFR/ERBB and down-regulation of ER signaling pathway, and this negative feedback regulation can impact the efficacy of CDK4/6 inhibitors (26) (**Figure 3**, **Table 2**).

### Other Mechanisms

#### EMT

Epithelial-mesenchymal transformation (EMT) is a biological process in which epithelial cells lose their polarity obtain the ability to invade and migrate. EMT has important roles in tumor cell metastasis, tumor stem cell formation, drug resistance, and other malignant phenotypes. A number of EMT-related signaling pathways are involved in drug resistance in cancer cells (83–85). The gene set enrichment analysis (GSEA) revealed enrichment of pathways that regulate EMT and cancer stem cells (IL-6/Stat3, IL-2/STAT-5, Notch, Wnt) in the cells resistant to palbociclib (10). Indeed, anti-CDK4/6 therapy can induce EMT and enhance cell invasion through activating TGF-β signaling (60, 86). It was suggested that EMT is an important determinant of success/failure of targeted therapies by interfering with the compensatory changes such as deregulation of CDK2 activity (87). Low cyclin D1 (CCND1) expression displays increased expression of EMT markers, increased migration of breast cancer cells and drug resistance (86) (**Figure 3**, **Table 2**).

#### Immune Mechanisms

CDK4/6 inhibitors not only induce tumor cell cycle arrest, but also promote anti-tumor immunity (88–90). In murine models of breast carcinoma, it was found that CDK4/6 inhibitors can activate tumor expression of endogenous retroviral elements that enhance tumor antigen presentation. CDK4/6 inhibitors also suppress the proliferation of suppressive regulatory T cells (Tregs) and enhance the cytotoxic T cell-mediated killing of tumor cells. It was also found that CDK4/6 inhibitors could promote anti-tumor immunity by phosphorylating NFAT4, a transcription factor of T cells, thereby increasing IL-2 levels (91). CDK4/6 inhibitors reduced the proliferation of T cells, but increased tumor infiltration and activation of effector T cells. In addition, CDK4/6 inhibition can augment the response to PD-1 blockade in multiple *in vivo* murine syngeneic tumor models (91). These studies provide a rationale for combining CDK4/6 inhibitors with immunotherapy to more effectively killing tumor cells and preventing drug resistance (**Figure 3**, **Table 2**).

#### Autophagy

Autophagy is a cellular process that eliminates the damaged or aged cells and is the key machinery for bulk degradation of superfluous or aberrant cytoplasmic components. Autophagy is a double-edged sword in drug sensitivity/drug resistance (92–94). Autophagy could elevate the maintenance of cancer stem cells which may enhance drug resistance, while autophagy may help tumor cells to clear the drug-induced damage which decreasing the impact of chemotherapy and enhances therapeutic response (95, 96). It was demonstrated that CDK4/6 inhibition induces ROS mediated senescence and autophagy, blockade of autophagy significantly improves the efficacy of CDK4/6 inhibition (10). It was reported that high expression of autophagy proteins like LC3B can be utilized to combat resistance to cell-cycle-targeted therapies, such as CDK4/6 inhibitors (94). More research is needed to clarify the relationship between the CDK4/6 inhibitor and autophagy, this will provide a better prospect for the clinical application (**Figure 3**, **Table 2**).

## Summary and Perspectives

CDK4/6 inhibitors are an effective therapeutic option for patients. A number of clinical trials have demonstrated the effectiveness and benefits of CDK4/6 inhibitors in improving the progression-free survival (PFS) of patients with ER-positive, HER2-negative advanced breast cancer (ABC) or metastatic breast cancer (MBC) when combined with endocrine therapy. The approval of palbociclib was based on the results from the PALOMA-1/TRIO-18, PALOMA-2, and PALOMA-3 trials. In the PALOMA-1 trail, combined therapy of letrozole with palbociclib significantly improved PFS as compared with single-agent letrozole. The PALOMA-2 trial confirmed the clinical activity of combination of palbociclib with letrozole. In PALOMA-3 trial, combined treatment of palbociclib with fulvestrant has shown benefits in patients with HR-positive, HER2-negative ABC or MBC. Thus, FDA approved the combined use of palbociclib with fulvestran based on this trial (3, 6, 21, 97). Abemaciclib was approved based on the results of MONARCH 1, MONARCH2, and MONARCH3, and combination of abemaciclib with fulvestrant has been approved for treatment of patients with HR-positive, HER2-negative ABC or MBC. MONARCH 3 trial showed that abemaciclib plus anastrozole or letrozole produced a significantly longer median PFS than the placebo plus anastrozole or letrozole. FDA has approved the combined therapy of abemaciclib in with an aromatase inhibitor as first-line treatment for postmenopausal women with HR-positive, HER2-negative ABC (8, 98, 99). In addition, ribociclib in combination with letrozole was approved as the first-line treatment for postmenopausal women with HR-positive and HER2-negative ABC or MBC, and the combination of ribociclib with fulvestrant was approved for the treatment of postmenopausal women with HR-positive and HER2-negative ABC, based on the outcomes from clinical trials. MONALEESA-7 trial compared patience received ovarian function suppression and endocrine therapy plus ribociclib or not, in the ribociclib group, the PFS and overall survival (OS) was significantly long than placebo group (100–102). The recent study SOLAR-1, indicated that when alpelisib was combined with fulvestrant to treat the patients with PIK3CA-mutated, HR+, HER2- ABC patients, the PFS was increased from 5.7 to 11.2 months, a statistically significant prolongation (103). In China, the CDK4/6 inhibitors have been introduced into the first-line treatment for patients with advanced estrogenic receptor positive breast cancer. While this new targeted therapy has benefited numerous patients with advanced breast cancer, drug resistance to CDK4/6 inhibitors remain to be a major impediment to successful treatment of the disease. Novel approaches to preventing or overcoming the resistance to CDK4/6 inhibitors would certainly increase the value and benefits of these agents to breast cancer patients. However, to reach this goal, we need to have a better understanding of the multiplicity and complexity of the molecular mechanisms involved in resistance to CDK4/6 inhibitors. Also, despite enormous advances in this targeted therapy in treating breast cancer, its clinical efficacy and benefits are limited by the patient populations that do not benefit from this remedy, and this might be associated with a variety of factors such as tumor heterogeneity and target alterations. Identification and development of predictive and reliable biomarkers for the response to CD4/6 inhibitors shall significantly improve the outcome and value of the CD4/6-targeted therapy through better selecting appropriate patients for specific therapeutic regimens, thus are urgently needed. With a better understanding of the molecular mechanism behind resistance to CDK4/6 inhibitors, we could anticipate that patients can better benefit from novel therapeutic strategies that prevent and circumvent drug resistance and reinforce the efficacy of this targeted therapy.

## Author Contributions

HJ contributed to the conception of the study. RL finished the first manuscript preparation. BW revised the manuscript. SW, XL, JR, JL, KB, YW helped perform the analysis with constructive discussions. All authors contributed to the article and approved the submitted version.

## Conflict of Interest

The authors declare that the research was conducted in the absence of any commercial or financial relationships that could be construed as a potential conflict of interest.
